# Efficacy of Modified Ban Xia Xie Xin Decoction on Functional Dyspepsia of Cold and Heat in Complexity Syndrome: A Randomized Controlled Trial

**DOI:** 10.1155/2013/812143

**Published:** 2013-03-17

**Authors:** Luqing Zhao, Shengsheng Zhang, Zhengfang Wang, Chuijie Wang, Suiping Huang, Hong Shen, Wei Wei, Hongbing Wang, Bing Wu

**Affiliations:** ^1^Department of Gastroenterology, Beijing Hospital of Traditional Chinese Medicine Affiliated to Capital Medical University, No. 23 Meishuguan Back Street, Dongcheng District, Beijing 100010, China; ^2^Department of Gastroenterology, The Affiliated Hospital of Liaoning University of Traditional Chinese Medicine, No. 33 Beiling Street, Huanggu District, Shenyang 110033, China; ^3^Department of Gastroenterology, The Second Affiliated Hospital of Guangdong University of Traditional Chinese Medicine, No. 111 Dade Street, Baiyun District, Guangzhou 510120, China; ^4^Department of Gastroenterology, The Affiliated Hospital of Nanjing University of Traditional Chinese Medicine, No. 155 Hanzhong Street, Jianye District, Nanjing 210029, China; ^5^Department of Gastroenterology, Beijing Xuanwu Hospital of Traditional Chinese Medicine, No. 8 Wanming Street, Xicheng District, Beijing 100102, China

## Abstract

*Background*. Chinese herbal medicine (CHM) has been used in China and elsewhere to treat patients with functional dyspepsia (FD). However, controlled studies supporting the efficacy of such treatment are lacking. *Objective*. To assess the efficacy and safety of modified Ban xia xie xin decoction in patients with FD of cold and heat in complexity syndrome. *Methods*. We performed a randomized, double-blind, placebo-controlled trial involving patients from five centers. Patients with FD of cold and heat in complexity syndrome (*n* = 101) were randomly assigned to groups given either CHM modified Ban Xia Xie Xin decoction or placebo in a 2 : 1 ratio. Herbal or placebo granules were dissolved in 300 mL of boiled water cooled to 70°C. Patients in both groups were administered 150 mL (50°C) twice daily. The trial included a 4-week treatment period and a 4-week followup period. The primary outcomes were dyspepsia symptom scores, measured by the total dyspepsia symptom scale and the single dyspepsia symptom scale at weeks 0, 1, 2, 3, 4, and 8. *Results*. Compared with patients in the placebo group, patients in the CHM group showed significant improvements according to the total and single dyspepsia symptom scores obtained from patients (*P* < 0.01) and investigators (*P* < 0.01). *Conclusions*. CHM modified Ban Xia Xie Xin decoction appears to offer symptomatic improvement in patients with FD of cold and heat in complexity syndrome. *Trial Registration*. Chinese Clinical Trial Registry (ChiCTR): ChiCTR-TRC-10001074.

## 1. Introduction

Functional dyspepsia (FD) is a common functional gastrointestinal disorder characterized by chronic or recurrent upper abdominal fullness, epigastric pain, eructation, bloating, early satiety, nausea, vomiting, regurgitation, burning, loss of appetite, and other symptoms. FD accounts for a significant proportion of patients seen in gastroenterology offices. The global prevalence of FD is estimated to be 11.5% to 29.2% [1–4]. The direct and indirect economic burden caused by FD is huge and has a considerable negative impact on productivity [5, 6]. The pathophysiology of FD is poorly understood, although various mechanisms are thought to play a role in the development of symptoms [7–10]. No single available treatment is reliably effective for this condition. Many studies have suggested the potential effectiveness of Chinese herbal medicine (CHM) in the treatment of FD [11]. Ban Xia Xie Xin decoction has been widely used for the treatment of patients with FD of cold and heat in complexity syndrome [12, 13]. However, most previous clinical trials have lacked rigor and used poor techniques for randomization and blinding. To date, relatively few multicenter, prospective, randomized, placebo-controlled, double-blind studies on using CHM to treat FD have been performed.

In Traditional Chinese Medicine (TCM), FD is considered to be nearly equivalent to the TCM term “stuffiness and fullness” [14], which is divided into different syndromes according to the clinical symptoms and signs. In our previous research, we studied the distribution of the different syndromes in 565 patients with FD and found that “cold and heat in complexity” is one of the most common syndromes of FD [15]. Ban Xia Xie Xin decoction is a traditional Chinese compound herbal recipe for mild regulation of cold and heat. We added related herbal medicines (Cortex Magnoliae officinalis, Medicated Leaven, Ark Shell) to that recipe to identify the formula of “modified Ban Xia Xie Xin decoction” that had a satisfactory clinical effect. Moreover, previous studies have shown that the active ingredients in the modified Ban Xia Xie Xin decoction can reinforce the protective function of the mucosa, regulate gastrointestinal function, and induce anti-inflammatory action against *Helicobacter pylori* [16–20].

In this trial, we tested the efficacy of the modified Ban Xia Xie Xin decoction in patients with FD and cold and heat in complexity syndrome using a randomized, double-blind, placebo-controlled study design.

## 2. Materials and Methods

### 2.1. Design

This study was a double-blind, placebo-controlled clinical trial. Patients were randomized into CHM or placebo groups in a 2 : 1 ratio. Because it would be unethical to assign an equal number of ill subjects to the ineffective placebo treatment, the 2 : 1 randomization plan was chosen to protect the rights of the subjects. The trial protocol was approved by regional ethics review boards, including the National Review Board for Clinical Drug Research in the Beijing Hospital of Chinese Medicine Hospital affiliated to Capital Medical University. There were no major changes in the study protocol after initiation of the study. 

### 2.2. Participants

Patients were screened by investigators at five sites in China: the Beijing Hospital of Traditional Chinese Medicine affiliated to Capital Medical University, the Affiliated Hospital of Liaoning University of Traditional Chinese Medicine, the Second Affiliated Hospital of Guangdong University of Traditional Chinese Medicine, the Affiliated Hospital of Nanjing University of Traditional Chinese Medicine, and the Beijing Xuanwu Hospital of Traditional Chinese Medicine. The study was conducted between April 2009 and March 2011. Patients were assessed according to the Rome III criteria and *The Guiding Principle for Clinical Research on New Drugs of Traditional Chinese Medicine* [14]. The inclusion and exclusion criteria are shown in Table 1. Written informed consent was obtained from all patients prior to inclusion in the trial. Patients were free to withdraw from the study at any time. 

### 2.3. Randomization and Blinding

Randomization was performed with SAS9.10 (block size 6). Patients and investigators were all blinded. Eligible patients were assigned a randomization number according to a predetermined list at each center. These numbers were allocated to patients in sequential order and registered in the patient enrolment list, and the allocation was concealed. Emergency envelopes containing the randomization code were provided to the investigators and were examined at the end of the trial to ensure that the blinded conditions had been maintained.

### 2.4. Interventions

Patients in the CHM group were provided granules of Chinese herbal extracts prepared by Tcmages Pharmaceutical Co., Ltd. (Beijing, China). The standard herb formula (Table 2) was a modified Ban Xia Xie Xin decoction. Patients in the placebo group were given placebo granules that had been prepared by the same supplier and were designed to taste, smell, and look similar to the Chinese herbal formula granules. To ensure that the patients were not able to discriminate between placebo and active treatments, 20 healthy volunteers participated in a randomized taste and visual assessment of the placebo and active medication. Eight volunteers correctly identified the active compound as active, whereas 12 volunteers considered the placebo preparation to be the active compound. Thus, it is reasonable to assume that the medication was given in an appropriately blinded manner. Granules were dissolved in 300 mL of boiled water cooled to 70°C. Patients in both groups were required to take 150 mL (50°C) twice daily. For the duration of the trial, the patients were not allowed to take any concomitant medications associated with the treatment of FD. Treatment continued for 4 weeks and was followed by a 4-week followup period. 

### 2.5. Outcomes

We assessed FD symptoms using two scales: (1) the total dyspepsia symptom (TDS) scale and (2) the single dyspepsia symptom (SDS) scale. Ratings were completed by both the investigators and patients at baseline and at weeks 1, 2, 3, 4, and 8.

#### 2.5.1. Total Dyspepsia Symptom Scale

The TDS scale assessed eight items (postprandial fullness and bloating, early satiety, epigastric pain, epigastric burning, nausea, vomiting, eructation, and “other symptoms”), each with four scoring options (absent = 0, mild = 1, moderate = 2, or severe = 3). The percentage of TDS score improvement was calculated using the following formula: (TDS score of week 0−TDS score of week 4)/TDS score of week 0.

#### 2.5.2. Single Dyspepsia Symptom Scale

The SDS scale measured three aspects of four principal symptoms of FD: epigastric pain, epigastric burning, postprandial fullness and bloating, and early satiety. The three aspects were frequency, intensity, and level of discomfort and were rated by four scoring options (absent = 0, mild = 1, moderate = 2, or severe = 3). The total score obtained using this scale was called the SDS score. The percentage of SDS score improvement was calculated using the following formula: SDS score of week 0−SDS score of week 4)/SDS score of week 0.

### 2.6. Safety Monitoring

To assess the safety of the 4-week treatment, routine blood, urine, and stool sample tests as well as electrocardiogram and blood biochemical tests (ALT, AST, BUN, and Scr levels) were conducted before randomization and immediately after the completed treatment. During the trial, adverse events were observed in detail and documented using case report forms.

### 2.7. Sample Size

We performed sample size calculations in two ways. To guarantee the reliability of the trial, the calculation yielding the larger sample size was used. The sample size was calculated according to the following formula [21]:

(1) n1=[uαπc(1−πc)(1+c)c   +  uβπ1(1−π1)+π2(1−π2)c]2  ×((π1−π2)2)−1,n2=cn1n1CHM, n2  placebo,πc=π1+cπ21+c, uα=1.64, uβ=1.28, c=2,              π1=0.5, π2=0.80.

The patients were assigned to either the CHM group or the placebo group (in a 2 : 1 ratio). The effective rates of treatment and placebo were assumed to be 80% and 50%, respectively [22, 23]. The calculation indicated that a sample size of 90 would be sufficient (*n* = 60 in the treatment group, *n* = 30 in control group). To allow for a 15% rate of dropouts and missing data, the sample size was 105 (*n* = 70 in the treatment group, *n* = 35 in control group). However, due to time limitations, we recruited 67 patients for the treatment group and 34 patients for the control group.

### 2.8. Statistical Analysis

We performed intention-to-treat analyses using all available data at each time point and the baseline-observation-carried-forward approach for missing data. The statistical analysis was performed by the Center of Clinical Epidemiology of the Third Hospital of Peking University. Parametric Student's *t*-tests or nonparametric Wilcoxon tests were used to quantitatively compare variables according to distribution characteristics. Quantitative variables are reported as mean ± SD. In this trial, there were two primary endpoints (TDS and SDS scores). Therefore, for multiple testing problems, the significance level underwent Bonferroni correction at *P* < 0.025. 

## 3. Results

### 3.1. Study Population

Between April 2009 and March 2011, a total of 101 patients were recruited; 67 were randomized into the CHM group and 34 into the placebo group. Ten patients withdrew from the trial due to a lack of efficacy. No serious adverse events were reported. The physiological tests obtained after 4 weeks of treatment showed no abnormal values.

### 3.2. Participant Flow

**Figure 1:**
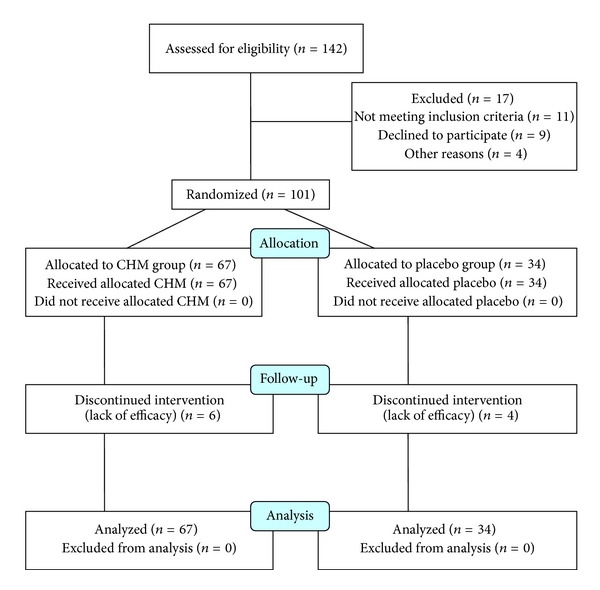
Flow of participants in the study.

The flow of participants in the study is summarized in Figure 1.

### 3.3. Baseline Data

The general characteristics of the patients are shown in Table 3. No significant differences were identified between the two groups in terms of parameters such as gender, age, course of disease, or symptom scores before treatment.

### 3.4. Primary Outcome Variables

#### 3.4.1. Total Dyspepsia Symptoms Scale Score

After 4 weeks of treatment, the TDS score assessed by investigators was significantly better for the CHM group than for the placebo group (*Z* = − 4.547, *P* < 0.01). At week 8, the score was also significantly better for CHM than for placebo (*Z* = − 3.878, *P* < 0.01). The TDS scores provided by the patients themselves were similar to those given by the investigators (Table 4). The percentage of TDS score improvement after 4 weeks of treatment is summarized in Table 5.

The results were clinically meaningful. Ratings of the clinical global impression of improvement after the treatment showed the following significant results for the treatment versus placebo group, respectively: very much improved (47.8% versus 5.9%), much improved (28.4% versus 26.5%), slightly improved (10.4% versus 23.5%), and unchanged or deteriorated (13.4% versus 44.1%) (*P* < 0.001).

#### 3.4.2. Single Dyspepsia Symptom Scale Score

SDS scores assessed by investigators. After 4 weeks of treatment, the scores of epigastric pain, postprandial fullness and bloating, early satiety, and burning sensation were significantly better for the CHM group than for placebo (*P* < 0.01). At week 8, the scores of epigastric pain, postprandial fullness and bloating, early satiety, and burning sensation were significantly better for CHM than for placebo (*P* < 0.01). 

The SDS scores provided by patients were similar to those given by investigators. The percentage of SDS score improvement after 4 weeks of treatment is summarized in Table 5.

## 4. Discussion

FD is a heterogeneous disorder. It involves many pathogenic factors and different pathophysiological disturbances, including delayed gastric emptying, impaired accommodation, and hypersensitivity to gastric distention. Treatment of the underlying pathophysiological abnormality seems logical, but the main pharmacotherapeutic options include acid suppression, prokinetic drugs, and antidepressants [6, 24–26], all of which have limited effects. Herbal formulations are widely used to treat FD in China and many other areas in the world. However, the available evidence of the efficacy of these formulas is inadequate. 

This multicenter, randomized, double-blind, placebo-controlled study indicated that modified Ban Xia Xie Xin decoction is effective in the management of symptoms associated with FD. The effects appeared to last for up to 4 weeks after completion of treatment and were particularly beneficial for epigastric pain, postprandial fullness and bloating, early satiety, and burning sensation. Patients treated with modified Ban Xia Xie Xin decoction demonstrated significantly better outcomes (both clinically and statistically) for all outcome measures compared with patients receiving placebo. Moreover, no serious adverse events were reported during the study.

The evaluation of treatment effects in patients with FD is difficult, and there is currently no gold standard. In our study, we used two different parameters as the target variables. The TDS scale included almost all symptoms associated with FD, and the SDS scale included information on the four principal symptoms of FD, measured in terms of the frequency, intensity, and level of discomfort. The target variables were recorded by both investigators and patients. Another difficulty in clinical trials involving patients with FD is the remarkable placebo response. It has been shown that one-third of patients with FD will respond to placebo in short-term trials [27], and the proportion may be even higher in long-term studies. In our study, we made a great effort to make the treatments in the two groups indistinguishable to the patients. A placebo of similar appearance, smell, and taste to the active concoction was used. To ensure that the patients were not able to discriminate between placebo and active treatment, 20 healthy volunteers participated in a randomized taste and visual assessment of the placebo and active medication. Eight volunteers correctly identified the active compound as active, whereas 12 volunteers considered the placebo preparation to be the active compound. Thus, it is reasonable to assume that the medication was given in an appropriately blinded manner. Despite the well-known high response rate to placebo in patients with FD, we found significantly greater improvements in dyspepsia symptoms in patients receiving the CHM compared with those receiving placebo.

In TCM, injury by food or drink, emotional injury, and congenital defects are the main pathogenic factors of FD. All pathogenic factors cause abnormal function of the upper abdominal spleen and stomach and the complexity of cold and heat. The herbal formula provided to patients in this study was a modified Ban Xia Xie Xin decoction. Ban Xia Xie Xin decoction is a traditional Chinese compound herbal recipe used to regulate cold and heat. We added related herbal medicines (Cortex Magnoliae officinalis, Medicated Leaven, Ark Shell) to the recipe to identify the formula of “modified Ban Xia Xie Xin decoction” that had a satisfactory clinical effect. All of the herbs matched well, so the complexity of cold and heat was regulated and the spleen-stomach function was recovered. Therefore, all dyspepsia symptoms would be abated. This is in accordance with previous studies that showed physiological effects of the active ingredients in the modified Ban Xia Xie Xin decoction. In China, Ban Xia Xie Xin decoction is used for FD, and some of the active ingredients in the modified Ban Xia Xie Xin decoction have been shown to reinforce the protective function of the mucosa, regulate gastrointestinal function, and induce anti-inflammatory action against *H. pylori* [16–20]. However, herbal preparations are complex and contain a number of active ingredients that may work together. The multiple effects of different active ingredients may be of benefit for the variety of different symptoms that occur in functional gastrointestinal disorders. However, more studies are needed to explore the mechanisms of action and properties of the identified components. FD is a common, chronic, and recurrent functional gastrointestinal disorder. This study used a short treatment period and followup and a relatively small number of patients; so, there is ample room to enhance the evaluation of efficacy and safety by further studies. 

## 5. Conclusions

We conclude that modified Ban Xia Xie Xin decoction may offer symptomatic improvements in patients with FD. In this randomized, double-blind, placebo-controlled trial, modified Ban Xia Xie Xin decoction was shown to be effective in the management of FD. Further studies are needed to determine the precise mechanisms of action.

## Figures and Tables

**Table 1:** Inclusion and exclusion criteria.

Inclusion criteria
(1) Patients who meet the Rome III diagnosis standard of functional dyspepsia.
(2) Patients who have cold and heat in complexity syndrome.
(3) Patients aged 18 to 65 without gender limitation.
(4) Singed the informed consent.
Exclusion criteria
(1) Patients who combined with GI ulcer, erosive gastritis, atrophic gastritis, severe dysplasia of gastric mucosa, or suspicious
malignant lesion.
(2) Patients who have overlap syndrome combined with gastroesophageal reflux disease or irritable bowel syndrome.
(3) Patients whose syndrome is difficult to differentiate.
(4) Patients who have connective tissue diseases, diabetes or other endocrine disease, climacteric syndrome, or severe diseases in heart,
liver, lung, kidney, or blood.
(5) Pregnant or lactating women. Disabled people.
(6) Patients with history of alcoholic or drug abuse.
(7) Patients who have allergic constitution or known to be allergic to the drug used in this trial.
(8) Patients who are involved in other trials.
(9) Patients with poor compliance or other reasons that the researcher considered not to be appropriate to participate in this trial.
(10) Patients with severe depression and have suicidal tendency.

**Table 2:** Chinese herb formula.

Chinese name	Pharmaceutical name	Powdered herb, %	Extraction yield, %
Ban Xia	Pinellia Tuber	9.1%	20%–30%
Huang Qin	Radix Scutellariae	9.1%	20%–30%
Huang Lian	Rhizoma Coptidis	4.5%	10%–20%
Gan Jiang	Dried Ginger	9.1%	10%–20%
Dang Shen	Pilose Asiabell Root	13.6%	40%–70%
Gan Cao	Liquorice Root	4.5%	20%–30%
Hou Po	Cortex Magnoliae Officinalis	9.1%	10%–20%
Shen Qu	Medicated Leaven	13.6%	20%–30%
Wa Lengzi	Ark Shell	27.3%	40%–70%

**Table 3:** Patient characteristics.

Variables	CHM (*n* = 67)	Placebo (*n* = 34)	*P* values
Characteristic			
Mean age ± SD, year	39.87 ± 12.89	40.50 ± 12.44	*P* > 0.05
Sex ratio (male : female)	21 : 46	13 : 21	*P* > 0.05
Mean height ± SD, cm	164.15 ± 8.27	165.15 ± 6.27	*P* > 0.05
Mean weight ± SD, kg	58.67 ± 10.79	60.47 ± 13.25	*P* > 0.05
Mean course of disease ± SD, month	46.67 ± 59.41	37.68 ± 38.73	*P* > 0.05

**Table 4:** TDS and SDS scores.

Variables	CHM (*n* = 67) Mean ± SD	Placebo (*n* = 34) Mean ± SD	*P* values
*Baseline date (week 0) *			
Gastroenterologist TDS scores	7.12 ± 2.71	7.68 ± 2.83	*P* > 0.05
Patient TDS scores	7.12 ± 2.69	7.59 ± 2.79	*P* > 0.05
Gastroenterologist SDS scores			
Epigastric pain	3.85 ± 2.18	3.47 ± 2.63	*P* > 0.05
Epigastric burning	2.36 ± 2.66	2.76 ± 2.63	*P* > 0.05
Postprandial fullness and bloating	4.96 ± 1.78	4.89 ± 2.05	*P* > 0.05
Early satiety	3.10 ± 2.32	3.32 ± 2.92	*P* > 0.05
Patient SDS scores			
Epigastric pain	3.90 ± 2.19	3.41 ± 2.64	*P* > 0.05
Epigastric burning	2.43 ± 2.68	2.76 ± 2.64	*P* > 0.05
Postprandial fullness and bloating	4.96 ± 1.78	4.88 ± 2.13	*P* > 0.05
Early satiety	3.09 ± 2.34	3.29 ± 2.94	*P* > 0.05
*Week 4 *			
Gastroenterologist TDS scores	2.37 ± 2.15	5.09 ± 3.00	*P* < 0.01
Patient TDS scores	2.43 ± 1.98	5.13 ± 3.32	*P* < 0.01
Gastroenterologist SDS scores			
Epigastric pain	1.22 ± 1.72	2.59 ± 2.38	*P* < 0.01
Epigastric burning	0.78 ± 1.55	2.47 ± 2.30	*P* < 0.01
Postprandial fullness and bloating	1.79 ± 1.99	3.32 ± 1.84	*P* < 0.01
Early satiety	0.76 ± 1.62	1.82 ± 2.05	*P* < 0.01
Patient SDS scores			
Epigastric pain	1.23 ± 1.76	2.46 ± 2.34	*P* < 0.01
Epigastric burning	0.78 ± 1.55	2.42 ± 2.67	*P* < 0.01
Postprandial fullness and bloating	1.73 ± 1.89	3.45 ± 1.97	*P* < 0.01
Early satiety	0.77 ± 1.64	1.79 ± 2.04	*P* < 0.01
*Week 8 *			
Gastroenterologist TDS scores	2.42 ± 2.75	4.41 ± 2.49	*P* < 0.01
Patient TDS scores	2.61 ± 2.15	4.31 ± 2.45	*P* < 0.01
Gastroenterologist SDS scores			
Epigastric pain	1.12 ± 1.57	2.35 ± 2.27	*P* < 0.01
Epigastric burning	0.73 ± 1.53	1.62 ± 2.00	*P* < 0.05
Postprandial fullness and bloating	1.75 ± 1.92	3.62 ± 1.79	*P* < 0.01
Early satiety	0.61 ± 1.48	1.47 ± 1.78	*P* < 0.01
Patient SDS scores			
Epigastric pain	1.17 ± 1.54	2.35 ± 2.17	*P* < 0.01
Epigastric burning	0.72 ± 1.54	1.64 ± 2.30	*P* < 0.05
Postprandial fullness and bloating	1.76 ± 1.90	3.62 ± 1.79	*P* < 0.01
Early satiety	0.60 ± 1.44	1.45 ± 1.79	*P* < 0.01

**Table 5:** Percentage of TDS and SDS score improvements after 4 weeks of treatment.

Variables	CHM (*n* = 67)	Placebo (*n* = 34)
Gastroenterologist TDS scores	66.7%	33.7%
Gastroenterologist SDS scores		
Epigastric pain	68.3%	25.4%
Epigastric burning	66.9%	10.5%
Postprandial fullness and bloating	63.9%	32.1%
Early satiety	75.5%	45.2%
Patient TDS scores	65.9%	32.4%
Patient SDS scores		
Epigastric pain	68.5%	27.9%
Epigastric burning	67.9%	12.3%
Postprandial fullness and bloating	65.1%	29.3%
Early satiety	75.1%	45.6%

## References

[1] Shaib Y, El-Serag HB (2004). The prevalence and risk factors of functional dyspepsia in a multiethnic population in the United States. *American Journal of Gastroenterology*.

[2] Bernersen B, Johnsen R, Straume B (1996). Non-ulcer dyspepsia and peptic ulcer: the distribution in a population and their relation to risk factors. *Gut*.

[3] Hirakawa K, Adachi K, Amano K (1999). Prevalence of non-ulcer dyspepsia in the Japanese population. *Journal of Gastroenterology and Hepatology*.

[4] Lu CL, Lang HC, Chang FY (2005). Prevalence and health/social impacts of functional dyspepsia in Taiwan: a study based on the Rome Criteria Questionnaire Survey assisted by endoscopic exclusion among a physical check-up population. *Scandinavian Journal of Gastroenterology*.

[5] Brook RA, Kleinman NL, Choung RS, Smeeding JE, Talley NJ (2011). Excess comorbidity prevalence and cost associated with functional dyspepsia in an employed population. *Digestive Diseases and Sciences*.

[6] Brook RA, Kleinman NL, Choung RS, Melkonian AK, Smeeding JE, Talley NJ (2010). Functional dyspepsia impacts absenteeism and direct and indirect costs. *Clinical Gastroenterology and Hepatology*.

[7] Tack J, Lee KJ (2005). Pathophysiology and treatment of functional dyspepsia. *Journal of Clinical Gastroenterology*.

[8] Lee KJ, Tack J (2010). Duodenal implications in the pathophysiology of functional dyspepsia. *Journal of Neurogastroenterology and Motility*.

[9] Zeng F, Qin W, Liang F (2011). Abnormal resting brain activity in patients with functional dyspepsia is related to symptom severity. *Gastroenterology*.

[10] Savarino E, Zentilin P, Dulbecco P, Malesci A, Savarino V (2011). The role of acid in functional dyspepsia. *American Journal of Gastroenterology*.

[11] Zhang SS, Su DM, Zhao LQ (2011). Systematic review on the efficacy of TCM in the treatment of functional dyspepsia. *Chinese Journal of Integrated Traditional and Western Medicine on Digestion*.

[12] Zhao YJ, Song QZ (2011). Efficacy of Ban xia xie xin decoction on 48 cases with functional dyspepsia of cold and heat in complexity syndrome. *Shanxi Journal of Traditional Chinese Medicine*.

[13] Min GB (2009). Efficacy of Ban xia xie xin decoction on functional dyspepsia of cold and heat in complexity syndrome. *Guangxi Journal of Traditional Chinese Medicine*.

[14] Zheng XY (2002). *Guiding Principle of Clinical Research on New Drugs of Chinese Medicine (Trial Implementation)*.

[15] Zhang SS, Chen Z, Xu WJ (2008). Study on distribution characteristic of syndrome of 565 cases of functional dyspepsia by twice differentiation of symptoms and signs based on the ‘cold, heat, deficiency, excess’. *China Journal of Traditional Chinese Medicine and Pharmacy*.

[16] Wang YN, Chen DX (2007). Effects of Ban xia xie xin decoction on the motility of isolated rat colon and dodecadactylon. *Chinese Journal of Integrative Medicine*.

[17] Zhou W, Yin KK, Wang SX (2007). Protective effect of Ban xia xie xin decoction and its active components of total saponins on gastric mucosa of mice with Helicobacter Pylori infection. *Journal of New Chinese Medicine*.

[18] Chan LW, Cheah ELC, Saw CLL, Weng W, Heng PWS (2008). Antimicrobial and antioxidant activities of Cortex Magnoliae Officinalis and some other medicinal plants commonly used in South-East Asia. *Chinese Medicine*.

[19] Li Y, Xu C, Zhang Q, Liu JY, Tan RX (2005). In vitro anti-Helicobacter pylori action of 30 Chinese herbal medicines used to treat ulcer diseases. *Journal of Ethnopharmacology*.

[20] Kong W, Wang J, Xiao X, Chen S, Yang M (2012). Evaluation of antibacterial effect and mode of Coptidis rhizoma by microcalorimetry coupled with chemometric techniques. *Analyst*.

[21] Liu XU (2003). Experimental design and data processing (fourth). *Chinese Journal of Difficult and Complicated Cases*.

[22] Xiao-Xia A (2006). *The Observation of Ban Xia Xie Xin Soup Clinical Effect in Treating the Felling of Fullness With Cold and Heat*.

[23] Holtmann G, Talley NJ, Liebregts T, Adam B, Parow C (2006). A placebo-controlled trial of itopride in functional dyspepsia. *The New England Journal of Medicine*.

[24] Oshima T, Miwa H (2006). Treatment of functional dyspepsia: where to go and what to do. *Journal of Gastroenterology*.

[25] Mönkemüller K, Malfertheiner P (2006). Drug treatment of functional dyspepsia. *World Journal of Gastroenterology*.

[26] Hojo M, Miwa H, Yokoyama T (2005). Treatment of functional dyspepsia with antianxiety or antidepressive agents: systematic review. *Journal of Gastroenterology*.

[27] Talley NJ, Locke GR, Lahr BD (2006). Predictors of the placebo response in functional dyspepsia. *Alimentary Pharmacology and Therapeutics*.

